# Treating Trigeminal Schwannoma through a Transorbital Approach: A Systematic Review

**DOI:** 10.3390/jcm13133701

**Published:** 2024-06-25

**Authors:** Matteo De Simone, Anis Choucha, Guillaume Dannhoff, Doo-Sik Kong, Cesare Zoia, Giorgio Iaconetta

**Affiliations:** 1 Department of Medicine, Surgery and Dentistry “Scuola Medica Salernitana”, University of Salerno, Via S. Allende, 84081 Baronissi, Italy; giaconetta@unisa.it; 2 BrainLab s.r.l., Mercato San Severino, 84085 Salerno, Italy; 3 Department of Neurosurgery, Aix Marseille University, APHM, UH Timone, 13005 Marseille, France; anis.c13@gmail.com; 4 Laboratory of Biomechanics and Application, UMRT24, Gustave Eiffel University, Aix Marseille University, 13005 Marseille, France; 5 Department of Neurosurgery, Strasbourg University Hospital, 67000 Strasbourg, France; guillaume.dannhoff@neurochirurgie.fr; 6 Department of Neurosurgery, Samsung Medical Center, School of Medicine, Sungkyunkwan University, Seoul 03063, Republic of Korea; neurokong@gmail.com; 7 UOC of Neurosurgery, Ospedale Moriggia Pelascini, Gravedona e Uniti, 22015 Gravedona, Italy; gioiaoffice@gmail.com; 8 Neurosurgery Unit, University Hospital “San Giovanni di Dio e Ruggi, D’Aragona”, 84131 Salerno, Italy

**Keywords:** skull base surgery, transorbital approach, ETOA, trigeminal schwannomas, Meckel’s cave, CN V

## Abstract

**Background**: Trigeminal schwannomas (TSs) are uncommon tumors found along any segment of the fifth cranial nerve (CN V). Typically located at the skull base, these benign tumors carry substantial morbidity due to the extent of traditional surgical methods. Minimally invasive endoscopic surgery such as the endoscopic transorbital approach (ETOA) presents promising new avenues for treatment, with the transorbital approach emerging as a potentially successful alternative. **Methods**: This review systematically assesses the application of the ETOA in treating TSs. PubMed, Ovid MEDLINE, and Embase were thoroughly searched for articles detailing the use of the ETOA in clinical case studies. The outcomes of interest encompassed epidemiological profiling, surgical results, and complication rates. **Results**: This study included 70 patients with TSs (from six studies), with 22 males (31.4%) and 58 females (68.6%). Patients averaged 55 years and were monitored for around 16.4 months (on average). In most tumors, the middle cranial fossa was involved to some degree. The majority (87.2%) were large (3–6 cm) and underwent gross total resection (GTR) or near-total resection in 87.2% of patients. Preoperatively, sensory alterations were common, along with proptosis, neuropathic pain, and diplopia. Postoperatively, complications included ptosis, diplopia, sensory impairment, corneal keratopathy, masticatory difficulty, and neuralgia. The pure ETOA was the primary surgical technique used in 90% of cases, with no recurrence observed during the follow-up period. **Conclusions**: Using the ETOA to treat TSs demonstrated an oncologic control rate of 87.2%. Postoperative complications including ptosis, diplopia, and sensory disturbances have been observed, but careful monitoring and management can mitigate these problems. The ETOA emerges as a viable surgical option, especially for tumors involving the middle cranial fossa, capable of adapting to individual patient needs and demonstrating efficacy in TS management.

## 1. Introduction

Schwannoma, a benign tumor originating from perineural Schwann cells, is commonly found in the head and neck region, accounting for about 25–45% of all variants. Typically, it manifests around the fourth decade of life [1]. Schwannomas can develop along any somatic or sympathetic nerve, characterized by their solitary nature and slow growth. They are typically painless and lack neurological symptoms, except in cases of schwannomatosis, a variant associated with the third form of neurofibromatosis [2].

Potentially, all cranial nerves covered by Schwan cells can give rise to schwannomas, although CN VIII is the one most typically involved [3]. Trigeminal schwannomas (TSs), conversely, are rare and constitute only 0.2 percent of all intracranial tumors, and 0.8–5% of intracranial schwannomas [4].

TSs occur on average around age 50 [4,5], except in cases where they are associated with neurofibromatosis. The average age of diagnosis in these cases is even about 26 years; however, it should be remembered that patients with this diagnosis are initiated into screening paths that allow a diagnosis much earlier than in a general setting. As evidence of this, the mean baseline size is only about 11 mm [6].

These tumors can often be effectively treated by complete surgical excision [7,8]. In addition to the patient’s general indication for surgery, it is essential then to choose which approach to use and with what intent of radicality [9].

**Figure 1:**
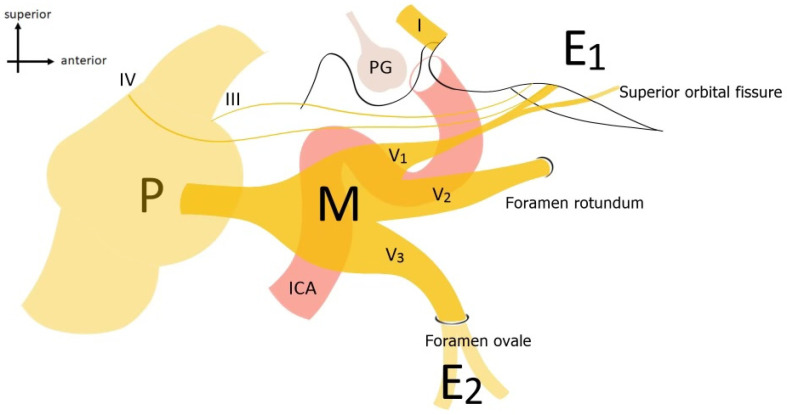
Anatomical outline of the CN V course with its anatomical relationships. P = Pons; M = Meckel’s cave; ophthalmic (V1), maxillary (V2), and mandibular (V3) branches; ICA = internal carotid artery; PG = pituitary gland.

All this complexity is functional for a thorough study of the topographic and functional anatomy of the fifth cranial nerve (CN V) in the skull base, an essential prerequisite for approaching this type of tumor safely and effectively. It is responsible for the sensation in the face, including touch, pain, and temperature. The nerve has three main branches: the ophthalmic nerve (V1), the maxillary nerve (V2), and the mandibular nerve (V3). The ophthalmic nerve innervates the upper face, including the forehead, scalp, and the front of the head. The maxillary nerve supplies sensation to the middle face, including the cheek, upper lip, and nasal cavity. The mandibular nerve provides sensation to the lower face, including the jaw, lower lip, and chin. Additionally, the trigeminal nerve plays a crucial role in controlling the muscles involved in chewing (mastication). It also has important connections with other cranial nerves, such as the facial nerve (VII), which controls facial expressions, and the glossopharyngeal nerve (IX), which is involved in taste sensation at the back of the tongue [10]. Below, Figure 1 schematizes in a lateral view the course of the fifth, in its cisternal portion, in its ganglionic portion received in Meckel’s cord, and of its branches.

Transcranial approaches have been described in the last century and can be schematized according to their trajectory into anterolateral, lateral, and posterolateral [11,12,13,14,15,16]. A synoptic description of their use is schematized in Table 1.

Advances in microsurgery, endoscopy, and overall skull base approaches have greatly improved treatment outcomes, with a high rate of complete resection. In addition, stereotactic radiosurgery, particularly Gamma Knife surgery (GKS), has emerged as an effective therapy for benign brain tumors [17]. A fortiori, a further advancement is endoscope-assisted skull base surgery. In particular, endoscopic transorbital approaches (ETOAs) are proposed as an attractive surgical solution to reach CN V regions in the middle and anterior cranial fossae [18]. However, studies comparing the different approaches in order to establish superiority in oncologic radicality and evaluating clinical outcomes, such as postoperative morbidity, are lacking. With this systematic literature review, we aim to comprehensively examine published case reports related to the treatment of trigeminal schwannomas (TSs) using the endoscopic transorbital approach (ETOA). Our goal is to outline the safety profile and oncologic efficacy of this approach, highlighting its limitations and strengths, and offering insights for structuring future clinical trials.

## 2. Materials and Methods

### 2.1. Literature Search

This systematic review is reported according to the Preferred Reporting Items for Systematic Reviews and Meta-Analysis (PRISMA) guidelines [19]. A comprehensive literature search of the databases PubMed, Ovid MEDLINE, and Ovid EMBASE was conducted. The search had no time limitations. The first literature search was performed on 18 March 2024, and the search was updated on 20 April 2024. A combination of keyword searches was performed to generate a search strategy. The search keywords, including “transorbital approach”, “Trigeminal schwannomas”, “skull base lesions”, and “Meckel’s cave’’ were used in both “AND” and “OR” combinations. Studies were found using the Medical Subject Heading (MeSH) terms and Boolean operators. Other pertinent articles were identified through a reference analysis of the selected papers. Only English-language articles were included. All studies were selected based on the following inclusion criteria: (1) case series or case reports, (2) lesions located in Meckel’s cave, (3) trigeminal schwannomas, and (4) the transorbital approach and its variations. The exclusion criteria were as follows: (1) meta-analyses and literature reviews, and (2) articles not reporting sufficient data on the presentation, diagnosis, or management. The list of identified studies was imported into Endnote X9, and duplicates were removed. The coauthors (M.D.S. and A.C.) independently performed the screening of abstracts for eligibility. Discordance between authors was resolved by the consensus of the senior authors (C.Z. and G.I.).

### 2.2. Risk of Bias Assessment

The Newcastle–Ottawa Scale (NOS) was used to assess the quality of the included studies [20]. A quality assessment was performed by assessing the selection criteria, the comparability of the study, and the outcome assessment [21]. The ideal score was 9. Higher scores indicated a better quality of studies. Studies receiving 7 or more points were considered high-quality studies. Two authors (M.D.S. and A.C.) performed the quality assessment independently. When discrepancies arose, papers were re-examined by a third author (C.Z.).

#### Statistical Analysis

Descriptive statistics are reported, including means and proportions. All analyses were performed with JMP 13.0 (www.jmp.com, (accessed on 12 April 20204), Cary, NC, USA). No formal statistical comparisons were performed due to the small sample sizes and insufficient power to detect differences between groups.

## 3. Results

### 3.1. Literature Review

**Figure 2:**
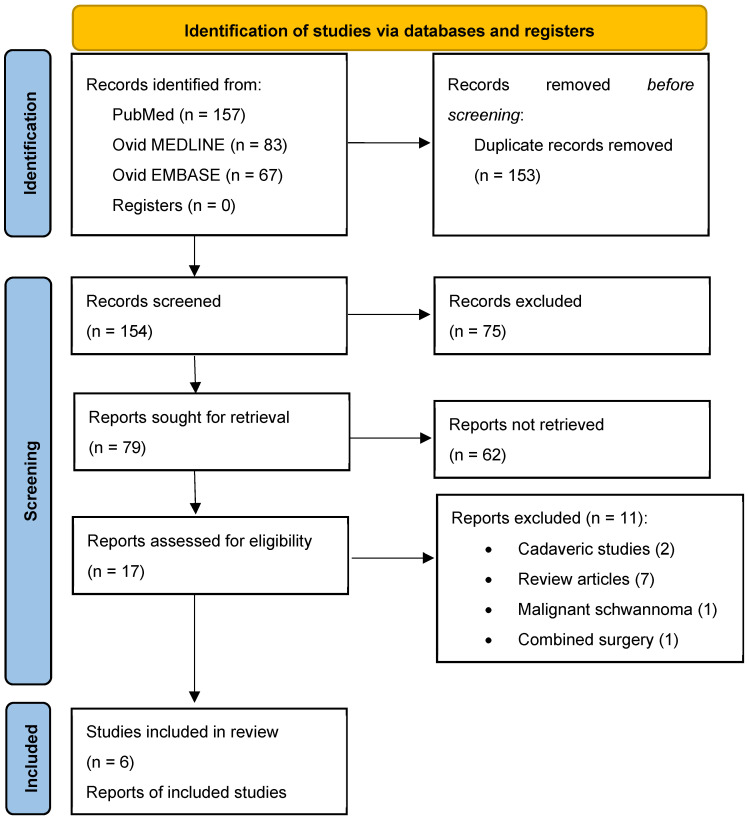
PRISMA flow diagram depicting the literature search process.

This review was registered in the Open Science Framework on the 29th of May 2024 (https://osf.io/mn2f7, accessed on 29 May 2024). IRB or ethical approval was not required for this study. The search strategy led to the identification of 154 studies from PubMed, 83 studies from Ovid MEDLINE, and 67 from Ovid EMBASE. Of these, 153 articles were duplicates. After removing the duplicates and applying the inclusion and exclusion criteria, we identified six papers about case series of patients with TSs treated with the ETOA published between 2019 and 2024 [18,22,23,24,25,26]. Figure 2 presents the PRISMA flowchart summarizing the identification, screening, eligibility, and inclusion criteria. An increasing trend in publications over recent years was observed.

### 3.2. Study Population and Clinical Data

The studies provided data on 70 patients diagnosed with TS. Among them, 22 were male, comprising 31.4% of the total, while the rest were female. On average, the patients in the cohort were 55 years old. They were monitored for an average duration of 16.4 months, though this varied considerably across studies (Table 2).

**Figure 3:**
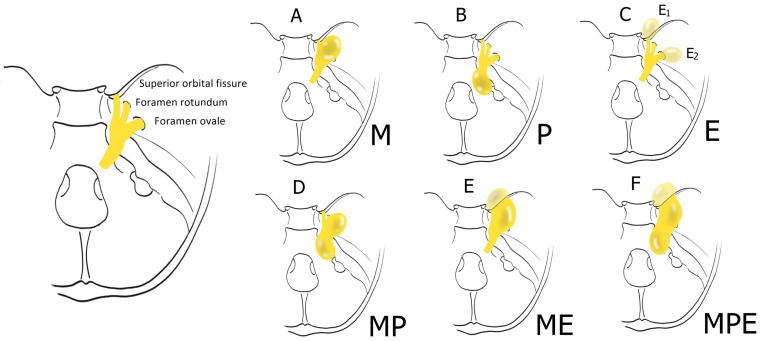
Yoshida and Kawase’s classification of TS.

Figure 3 visually represents these findings for each study. According to Yoshida and Kawase’s classification [27], TSs are classified into six types according to their location. Types M, P, and E are tumors involving a single compartment, i.e., the middle fossa, posterior fossa, or extracranial space, respectively. Types MP (middle and posterior fossa), ME (middle fossa and extracranial space), and MPE (middle and posterior fossa and extracranial space) are tumors involving multiple compartments.

In this case series that employed the ETOA, 24 cases were type M (34.3%), 28 cases were type MP (40%), 11 also had an extracranial exemption (MPE) (15.7%), 5 were type ME (7.1%), 2 were type E (2.9%), and none were type P.

In terms of size, most of these tumors, 61/70 (87.2%), were of large size, i.e., between 3 and 6 cm. Five (7.1%), on the other hand, had a major diameter greater than 6 cm, so they were defined as giant TSs, and only four (5.7%) were smaller than 3 cm, as shown in Table 3.

**Figure 4:**
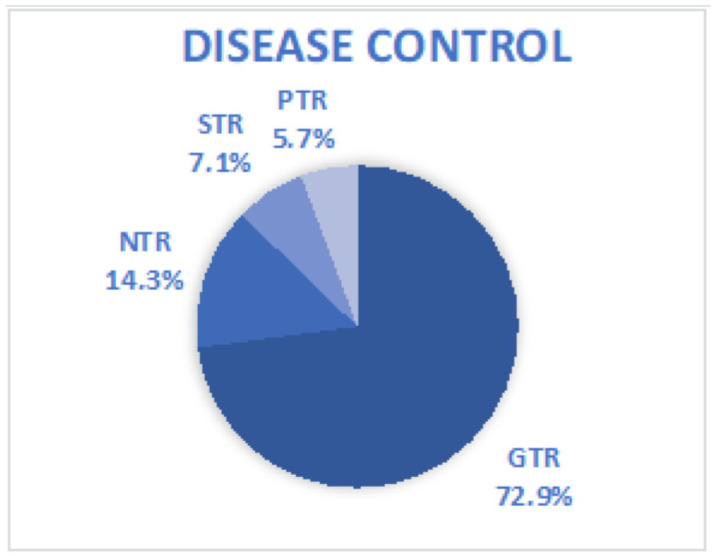
A pie chart schematizing the EOR of all cases considered in this review.

From the perspective of extent of resection (EOR), in 51 patients (72.9%), gross total resection (GTR) was achieved. If we add the 10 patients who achieved near-total resection (NTR) (14.3%), we can say that the percentage of patients who achieved good oncologic control of their disease rises to 87.2%. Finally, suboptimal disease control occurred in five cases (7.1%) in which sub-total resection (STR) was achieved, and in an additional four cases (5.7%), at least one of which was intentional, partial total removal (PTR) was achieved (Figure 4).

Regarding preoperative symptoms, most patients 54/70 (77.1%) experienced sensory alterations in the trigeminal area, such as facial numbness. Other frequent signs and symptoms were proptosis (11 cases—15.7%), neuropathic pain (7 cases—10%), and diplopia (19 cases—27.1%). Three of the patients in this case series presented as asymptomatic. Regarding postoperative signs and symptoms, there were six cases of ptosis, of which two were permanent. Ten patients had diplopia, of which three were permanent. A form of sensory impairment in the trigeminal innervation area was observed in six patients. Also, regarding ocular complications, seven patients reported corneal keratopathy, and in one case massive edema of the upper eyelid was observed. Finally, in 5 cases there was difficulty in chewing, and 14 had neuralgia with the need for medication.

Regarding the surgical technique, in 63 cases (90%) a pure ETOA was used. In three cases, an extended ETOA was performed. In an additional four cases, the ETOA was combined with an endoscopic endonasal EEA approach in two patients, and with a retrosigmoid lateral suboccipital (RSL) approach and suboccipital craniotomy (SOC) in the other 2. No recurrence was observed during the follow-up period (Table 4).

## 4. Discussion

Cushing and Eisenhardt viewed the treatment and prognosis of TS with pessimism, although they recognized that someday there might be a chance to approach these tumors safely and effectively [28]. Since they wrote these remarks in 1938, a long way has been traveled, and nevertheless no forecast has ever been so accurate.

Since Krayenbuhl’s first surgical series on two patients with trigeminal schwannomas in 1959 [29], there has been a proliferation of surgical cohort series. Initially, these series competed to reduce mortality, a goal facilitated by the advent of microsurgery in the 1970s, notably advanced by Yasargil [30,31,32,33]. Recent decades have seen series achieving 0% mortality, realizing Cushing’s vision of safely resecting lesions involving Meckel’s cave [34,35]. Consequently, the focus has gradually shifted toward quality of life through various strategies. Some have advocated for preplanned sub-total tumor resection to minimize iatrogenic damage to surrounding neurovascular structures, thereby preserving functional prognosis, followed by stereotactic radiosurgery on the residual tumor to match the oncological outcomes of total resection. Others have developed additional surgical approaches [36,37]. Finally, further advancements have been made in minimally invasive techniques such as endoscopic approaches, embodying the principle of “doing more with less” to spare anatomical structures and minimize collateral damage.

In fact, several transcranial microsurgical approaches (MTAs) to Meckel’s cave have been described over time, but there is still a lack of consensus among authors as to which approach can quantitatively offer the best exposure. As there is therefore no gold standard yet, the decision on the surgical approach will be relegated to the surgeon’s choice or surgical intent (total removal, or intentionally suboptimal, etc.). A recent cadaveric study provided a quantitative assessment of Meckel’s cave exposure across various MTAs [34].

The initial patients with TSs operated on through an endoscopic approach were treated via the endonasal route. Given that this approach was well studied and developed for addressing lesions such as pituitary adenomas, it was a natural progression to start this way. In their series on trigeminal schwannomas, Kassam et al. were the first to report a series of patients treated with the endoscopic endonasal approach in the middle cranial fossa [35]. Subsequently, others like Raza et al. and Shin et al. [36,37] have also shared their series, though they remain limited in number. They observed that lesions confined to the middle cranial fossa could benefit from an extended endoscopic endonasal approach with satisfactory outcomes, but lesions extending into the posterior cranial fossa might require a second-stage surgical intervention via a complementary retrosigmoid approach. More recently, Yin et al., with a series of 93 patients, expanded the indications for the endoscopic endonasal route to certain extracranial TSs [33].

As is well known, schwannomas involving the trigeminal branches and extending into the pterygopalatine or infratemporal fossae represent a surgical challenge. In these cases [34], the most effective surgical approach according to this study appears to be the endoscopic endonasal transpterygoid approach (EETPA). This approach offered a surface exposure of 73.9% for the medial part of V1, 91.3% for V2, and 50.3% for V3. It also provided good access to the Gasserion ganglion (GG) with a surface exposure of 47.4%. The EETPA also provides direct access to the pterygopalatine and infratemporal fossa. The ETOA in this same surgical study offered extensive exposure of the lateral portions of V1 and V3, with rates reaching 68.2% and 53.7%, respectively. However, the authors’ opinion on the exclusive use of this technique in the treatment of TSs appeared to be not so significant.

Indeed, in this study and others [38,39], the focus has traditionally been on extension along the coronal plane (latero-lateral direction). However, an evaluation of the results of this systematic review and the possibility of using the extended anterior endoscopic approaches (EEA and ETOA), or even these approaches used in combination, reveals that the real limitation lies rather in the sagittal trajectory; that is, in the anteroposterior direction. In particular, as this has evolved, it is evident how the ETOA, originally seen as a supplementary option to conventional transcranial/transnasal routes, has developed into established surgical approaches aimed at resolving the limitations of conventional procedures for specific skull base conditions. This can be achieved either independently or in combination with other approaches [40,41].

In our study, we found that a vast majority of the lesions were large TSs, measuring between 3 and 6 cm. This patient population resembles those in major microsurgical series, with the exception that we observed fewer giant schwannomas (larger than 6 cm). Given that endoscopic approaches involve a steep learning curve and potentially less maneuverability compared to more traditional microsurgical techniques, it is reasonable to infer that patients with giant trigeminal schwannomas were not included in these endoscopic series. These giant lesions can become infiltrative and also alter local anatomy (through bone erosion or displacement of neurovascular structures), meaning our systematic review cannot conclusively address or provide substantial insights specifically regarding these giant tumors. Moreover, our review also notes a very low incidence of small schwannomas (less than 3 cm). This could be explained by two factors: schwannomas are insidiously growing lesions, typically becoming clinically evident only once they reach a certain size with a mass effect, thereby reducing the prevalence of smaller diagnosed and hence treated schwannomas; secondly, such lesions are often asymptomatic at smaller sizes and are thus potentially eligible for stereotactic radiosurgery or simple monitoring.

In our study, we report a 70% rate of gross total resection, which aligns with findings from microsurgical series such as those by Wanibuchi and Fukushima [42]. However, more recent series, like that reported by Li et al., document over 90% gross total resection [31]. This is encouraging, considering that many centers are still early in their learning curve, with expected improvements in the coming years. Additionally, several studies have demonstrated that upfront stereotactic radiosurgery on residual schwannomas provides satisfactory oncological control. This suggests that this surgical approach is viable.

The mean follow-up was relatively low, i.e., 16.4 months (2 to 61 months). This prevents us from drawing conclusions about oncologic prognosis and the risk of lesion recurrence or regrowth. Thus, to infer oncologic prognosis, we rely solely on the extent of resection rates, as these are strongly associated with subsequent disease progression.

Regarding functional prognosis and the evolution of neurological deficits, the outcomes highlighted in this systematic review are seemingly comparable to those found in microsurgical series studies. Indeed, there is a tendency for improvement in facial neuralgia and a general persistence of facial hypesthesia. This becomes clear when a comparison is made with particularly large microsurgical case series [42]. However, it is important to note complications specific to this surgical approach, such as increased occurrences of ptosis, several corneal keratopathies, upper eyelid edema, and potentially more diplopia, though this requires confirmation in a future meta-analysis comparing these two surgical techniques. Finally, in the future, it will be useful to stratify these data according to the specific type of approach; in fact, remember that it is possible to conduct this approach by endoscope, exoscope, or open microsurgical techniques. Each of these approaches can potentially be conducted through four incisions, of which undoubtedly the most widely used by neurosurgeons is the Superior Eyelid Crease [43].

## 5. Limitations

This study is subject to the following shortcomings: (1) the retrospective nature of the available studies affects the overall quality of the evidence; (2) the small sample size of the population limiting generalizations; (3) two studies within the included set are from the same surgical group, potentially biasing the results; (4) the surgical series reflects the findings of internationally recognized experts in ETOAs, limiting generalizability; and (5) complications may have overlapping causes between the approach and location-related morbidity, complicating the specific determination of neurologic morbidity. However, we can say that the overall quality of the evidence is modest and that our study undoubtedly has the merit of systematizing the current literature, offering a scaffold on which to build prospective clinical trials, which will increase the overall quality of the evidence, establishing in which settings ETOA makes its greatest contribution definitively, always, and above all in the interest of the sufferers. In addition, given the relatively recent development of the approach, and the continued expansion of surgical indications, it is clear that limitations may arise in relation to the learning curve, which is why an individualized path is always suggested and especially that the surgeon compares with an adequate number of cases per year.

## 6. Conclusions

This study’s results underscore the efficacy of the ETOA in the treatment of TSs. With a high observed GTR or NTR rate of 87.2%, the ETOA emerges as a promising surgical option to achieve complete and safe tumor removal, especially when the large component is located at the level of the middle cranial fossa in selected patients. However, postoperative complications such as ptosis, diplopia, and sensory disturbances underscore the need for careful monitoring and postoperative management to ensure optimal patient outcomes and quality of life. The demographic and clinical profiles of TS patients reveal that sensory changes in the trigeminal area are the most prevalent preoperative symptom. In addition, the exploration of different surgical techniques, mainly a pure ETOA with some variations, underscores the adaptability of the ETOA to individual patient needs and tumor characteristics. In addition, the absence of recurrence during the follow-up period suggests the long-term efficacy and durability of the ETOA in treating TSs. However, longer-term studies with extended follow-up periods are needed to validate these findings and refine surgical techniques to improve patient outcomes.

## Figures and Tables

**Table 1:** Synoptic description of transcranial approaches to Mackel’s cave.

Category	Approach	Description
Anterolateral	Pterional Approach (PTA)	Described by Yasargil et al. [11], provides access to the anterolateral region of Meckel’s cave.
Anterolateral	Fronto-temporal-orbito-zygomatic Approach (FTOZA)	Following Van Furth et al. [12], allows wide lateral exposure of the skull and the ophthalmic nerve (V1).
Lateral	Kawase Approach (KWA)	Proposed by Kawase et al. [13], offers extensive lateral exposure of the trigeminal nerve.
Lateral	Subtemporal Approach (STA)	Based on Dolenc et al. [14], provides lateral access to the posterolateral region of Meckel’s cave.
Posterolateral	Retrosigmoid Approach (RSA)	According to Samii et al. [15], offers posterolateral access to the trigeminal nerve and the Gasserian ganglion.
Posterolateral	Retrosigmoid Approach with Suprameatal Extension (RSAS)	Following Samii et al. [16], provides wide posterolateral exposure of the trigeminal nerve trunk. Best for tumors in the cerebellopontine angle extending toward the Meckel cave and supratentorial regions.

**Table 2:** Studies selected and baseline information.

Study	Year	Study’s Nature	Case Number	Age (Mean)	Sex	Follow-Up (m)
Jeon et al. [18]	2019	Retrospective interventional case series	4	59.5 (52–66)	3F 1M	24
Park et al. [22]	2020	Retrospective multicenter	12	45.7 (21–68)	7F 5M	NA
Jeon et al. [23]	2021	Retrospective case series	1	66	1F	28
Han et al. [24]	2023	Retrospective case series	1	40	1F	6
Doo-Sik Kong et al. [25]	2023	Retrospective	50	46.9	35F 15M	21.9 (range 1–61.7)
Di Somma et al. [26]	2024	Retrospective (consecutive cohort)	2	56	1F 1M	2
Overall			70	55	48F 22M	

**Table 3:** Location and size of TSs.

Author, Year	Location							Size		
	M	P	MP	E	ME	MPE	Total	<3 cm	3–6 cm	>6 cm
Jeon et al., 2019 [18]	1	0	1	0	1	1	4	0	3	1
Park et al., 2020 [22]	4	0	5	2	1	0	12	3	5	4
Jeon et al., 2021 [23]	1	0	0	0	0	0	1	0	1	0
Han et al., 2023 [24]	0	0	1	0	0	0	1	0	1	0
Doo-Sik Kong et al., 2023 [25]	17	0	20	0	3	10	50	-	50 (average 3.11)	-
Di Somma et al., 2024 [26]	1	0	1	0	0	0	2	1	1	0
Overall	24	0	28	2	5	11	70	4	61 (87.2%)	5

Type M = middle fossa tumor in intradural space; type P = posterior fossa tumor in subdural space; type E = extracranial tumor in epidural space; Type MP = middle and posterior fossa; type ME = middle fossa and extracranial space; type MPE = middle and posterior fossa and extracranial space.

**Table 4:** Studies selected and clinical and surgical data.

Study	EOR	Preoperative Symptoms	Case Number	Postoperative Symptoms	Access (Intradural vs. Extradural)	Surgical Approach	Recurrency
Jeon et al., 2019 [18]	3 GTR 1 Intended PTR	1 Proptosis 2 Sensory changes (facial numbness)	4	1 complete ptosis	2 vs. 2	2 ETOA 1 ETOA plus SOC 1 extended ETOA	0
Park et al., 2020 [22]	9 GTR 1 NTR 2 STR	9 Sensory changes (numbness) 3 Proptosis 1 Diplopia	12	5 Sensory changes (numbness) 1 Diplopia medial gaze	10 vs. 2	11 ETOA 1 ETOA plus RLS	0
Jeon et al., 2021 [23]	1 GTR	Proptosis	1	Sensory changes (Transient numbness)	1 vs. 0	ETOA	0
Han et al., 2023 [24]	1 GTR	Facial numbness	1	None	NA	Extended ETOA	
Doo-Sik Kong et al., 2023 [25]	35 GTR 9 NTR 3 STR 3 PTR	42 Sensory changes 18 Diplopia 5 Proptosis 7 Neuralgic pain	50	7/1 Diplopia (transient/permanent) 4/1 Ptosis (transient/permanent) 2 Wound infection 7 Corneal keratopathy 5 Mastication difficulty 14 Neuralgia requiring medication	NA	47 ETOA 2 ETOA plus EEA 1 extended ETOA (lateral canthotomy)	0
Di Somma et al., 2024 [26]	2 GTR	None	2	Upper eyelid edema, diplopia lateral gaze,	NA	2 ETOA	0
				sensory changes (V2–3 numbness)			

EOR = extent of resection; GTR = gross total resection; PTR = partial tumor resection; NTR = near-total resection; STR = sub-total resection; RLS = retrosigmoid lateral suboccipital.

## References

[1] Argenyi Z.B., Cooper P.H., Santa Cruz D. (1993). Plexiform and other unusual variants of palisaded encapsulated neuroma. J. Cutan. Pathol..

[2] MacCollin M., Woodfin W., Kronn D., Short M.P. (1996). Schwannomatosis: A clinical and pathologic study. Neurology.

[3] Greene J., Al-Dhahir M.A. (2024). Acoustic Neuroma. 2023 Aug 17. StatPearls [Internet].

[4] Agarwal A. (2015). Intracranial trigeminal schwannoma. Neuroradiol. J..

[5] Aftahy A.K., Groll M., Barz M., Wagner A., Lange N., Butenschön V.M., Delbridge C., Bernhardt D., Meyer B., Negwer C. (2021). Surgical Outcome of Trigeminal Schwannomas. Cancers.

[6] Moualed D., Wong J., Thomas O., Heal C., Saqib R., Choi C., Lloyd S., Rutherford S., Stapleton E., Hammerbeck-Ward C. (2022). Prevalence and natural history of schwannomas in neurofibromatosis type 2 (NF2): The influence of pathogenic variants. Eur. J. Hum. Genet. EJHG.

[7] Ramina R., Mattei T.A., Sória M.G., da Silva E.B., Leal A.G., Neto M.C., Fernandes Y.B. (2008). Surgical management of trigeminal schwannomas. Neurosurg. Focus.

[8] De Simone M., Conti V., Palermo G., De Maria L., Iaconetta G. (2023). Advancements in Glioma Care: Focus on Emerging Neurosurgical Techniques. Biomedicines.

[9] De Simone M., Fontanella M.M., Choucha A., Schaller K., Machi P., Lanzino G., Bijlenga P., Kurz F.T., Lövblad K.-O., De Maria L. (2024). Current and Future Applications of Arterial Spin Labeling MRI in Cerebral Arteriovenous Malformations. Biomedicines.

[10] Joo W., Yoshioka F., Funaki T., Mizokami K., Rhoton A.L. (2014). Microsurgical anatomy of the trigeminal nerve. Clin. Anat..

[11] Yasargil M.G. (1976). Microsurgical pterional approach to the aneurysms of the basilar bifurcation. Surg. Neurol..

[12] van Furth W.R., Agur A.M.R., Woolridge N., Cusimano M.D. (2006). The Orbitozygomatic Approach. Neurosurgery.

[13] Kawase T., Toya S., Shiobara R., Mine T. (1985). Transpetrosal Approach for Aneurysms of the Lower Basilar Artery. J. Neurosurg..

[14] Dolenc V. (1983). Direct Microsurgical Repair of Intracavernous Vascular Lesions. J. Neurosurg..

[15] Samii M., Metwali H., Samii A., Gerganov V. (2013). Retrosigmoid Intradural Inframeatal Approach: Indications and Technique. Neurosurgery.

[16] Samii A., Giordano M., Samii M. (2023). Retrosigmoid Intradural Suprameatal Approach: 2-Dimensional Operative Video. Oper. Neurosurg..

[17] Peciu-Florianu I., Régis J., Levivier M., Dedeciusova M., Reyns N., Tuleasca C. (2021). Tumor control and trigeminal dysfunction improvement after stereotactic radiosurgery for trigeminal schwannomas: A systematic review and meta-analysis. Neurosurg. Rev..

[18] Jeon C., Hong C.K., Woo K.I., Hong S.D., Nam D.H., Lee J.I., Choi J.W., Seol H.J., Kong D.S. (2018). Endoscopic transorbital surgery for Meckel’s cave and middle cranial fossa tumors: Surgical technique and early results. J. Neurosurg..

[19] Page M.J., McKenzie J.E., Bossuyt P.M., Boutron I., Hoffmann T.C., Mulrow C.D., Shamseer L., Tetzlaff J.M., Akl E.A., Brennan S.E. (2021). The PRISMA 2020 statement: An updated guideline for reporting systematic reviews. BMJ.

[20] Stang A. (2010). Critical evaluation of the Newcastle-Ottawa scale for the assessment of the quality of nonrandomized studies in meta-analyses. Eur. J. Epidemiol..

[21] Luchini C., Stubbs B., Solmi M., Veronese N. (2017). Assessing the quality of studies in meta-analyses: Advantages and limitations of the Newcastle Ottawa Scale. World J. Meta-Anal..

[22] Park H.H., Hong S.D., Kim Y.H., Hong C.K., Woo K.I., Yun I.S., Kong D.S. (2020). Endoscopic transorbital and endonasal approach for trigeminal schwannomas: A retrospective multicenter analysis (KOSEN-005). J. Neurosurg..

[23] Jeon C., Hong S.D., Woo K.I., Seol H.J., Nam D.H., Lee J.I., Kong D.S. (2020). Use of endoscopic transorbital and endonasal approaches for 360° circumferential access to orbital tumors. J. Neurosurg..

[24] Han X., Yang H., Wang Z., Li L., Li C., Han S., Wu A. (2023). Endoscopic transorbital approach for skull base lesions: A report of 16 clinical cases. Neurosurg. Rev..

[25] Kong D.S., Kim Y.H., Lee W.J., Kim Y.H., Hong C.K. (2022). Indications and outcomes of endoscopic transorbital surgery for trigeminal schwannoma based on tumor classification: A multicenter study with 50 cases. J. Neurosurg..

[26] Di Somma A., Guizzardi G., Sanchez España J.C., Matas Fassi J., Topczewski T.E., Ferres A., Mosteiro A., Reyes L., Tercero J., Lopez M. (2024). Complications of the Superior Eyelid Endoscopic Transorbital Approach to the Skull Base: Preliminary Experience With Specific Focus on Orbital Outcome. J. Neuroophthalmol..

[27] Yoshida K., Kawase T. (1999). Trigeminal neurinomas extending into multiple fossae: Surgical methods and review of the literature. J. Neurosurg..

[28] (1938). Meningiomas. Their Classification, Regional Behaviour, Life History, and Surgical End Results. Bull. Med. Libr. Assoc..

[29] Krayenbuhl H. (1959). Das Neurinom des Nervus trigeminus [Neurinoma of tirgeminal nerve]. Bull. Schweiz. Akad. Med. Wiss..

[30] Samii M., Migliori M.M., Tatagiba M., Babu R. (1995). Surgical treatment of trigeminal schwannomas. J. Neurosurg..

[31] Li M., Wang X., Chen G., Liang J., Guo H., Song G., Bao Y. (2021). Trigeminal schwannoma: A single-center experience with 43 cases and review of literature. Br. J. Neurosurg..

[32] Sindou M., Pelissou I., Dolenc V.V. (1987). Trigeminal neurinomas. A special type of cavernous sinus tumors. The Cavernous Sinus.

[33] Yin J., Wu Y., Zhang Z., Zhang Y., He J., Yang Z., Wang B., Wang X., Liu G., Bie Z. (2023). Operative management of trigeminal schwannomas: Based on a modified classification in a study of 93 cases. Acta Neurochir..

[34] Zanin L., Agosti E., Ebner F., de Maria L., Belotti F., Buffoli B., Rezzani R., Hirt B., Ravanelli M., Ius T. (2023). Quantitative Anatomical Comparison of Surgical Approaches to Meckel’s Cave. J. Clin. Med..

[35] Kassam A.B., Prevedello D.M., Carrau R.L., Snyderman C.H., Gardner P., Osawa S., Seker A., Rhoton A.L. (2009). The front door to meckel’s cave: An anteromedial corridor via expanded endoscopic endonasal approach—Technical considerations and clinical series. Oper. Neurosurg..

[36] Shin S.S., Gardner P.A., Stefko S.T., Madhok R., Fernandez-Miranda J.C., Snyderman C.H. (2011). Endoscopic Endonasal Approach for Nonvestibular Schwannomas. Neurosurgery.

[37] Raza S.M., Donaldson A.M., Mehta A., Tsiouris A.J., Anand V.K., Schwartz T.H. (2014). Surgical management of trigeminal schwannomas: Defining the role for endoscopic endonasal approaches. FOC.

[38] Van Rompaey J., Bush C., Khabbaz E., Vender J., Panizza B., Solares C.A. (2013). What is the Best Route to the Meckel Cave? Anatomical Comparison between the Endoscopic Endonasal Approach and a Lateral Approach. J. Neurol. Surg. Part B Skull Base.

[39] Van Rompaey J., Suruliraj A., Carrau R., Panizza B., Solares C.A. (2014). Meckel’s cave access: Anatomic study comparing the endoscopic transantral and endonasal approaches. Eur. Arch. Otorhinolaryngol..

[40] Moe K.S., Bergeron C.M., Ellenbogen R.G. (2010). Transorbital neuroendoscopic surgery. Neurosurgery.

[41] Dallan I., Castelnuovo P., Locatelli D., Turri-Zanoni M., AlQahtani A., Battaglia P., Hirt B., Sellari-Franceschini S. (2015). Multiportal Combined Transorbital Transnasal Endoscopic Approach for the Management of Selected Skull Base Lesions: Preliminary Experience. World Neurosurg..

[42] Wanibuchi M., Fukushima T., Zomordi A.R., Nonaka Y., Friedman A.H. (2012). Trigeminal Schwannomas: Skull Base Approaches and Operative Results in 105 Patients. Oper. Neurosurg..

[43] De Simone M., Zoia C., Choucha A., Kong D.S., De Maria L. (2024). The Transorbital Approach: A Comprehensive Review of Targets, Surgical Techniques, and Multiportal Variants. J. Clin. Med..

